# "Bong lung" in cystic fibrosis: a case report

**DOI:** 10.1186/1752-1947-4-371

**Published:** 2010-11-19

**Authors:** Zoe Gao, Richard Wood-Baker, Robin Harle, Kon Muller, Jenny Hauser, David W Reid

**Affiliations:** 1 Departments of Respiratory Medicine, Royal Hobart Hospital, Liverpool Street, Hobart, Tasmania, 7000, Australia; 2 Radiology, Royal Hobart Hospital, Liverpool Street, Hobart, Tasmania, 7000, Australia; 3 Discipline of Pathology, University of Tasmania Medical School, Collins Street, Hobart, Tasmania, 7000, Australia

## Abstract

**Introduction:**

Marijuana or "bong" lung has been recently described. Subjects typically develop large peripheral paraseptal lung bullae and are predisposed to spontaneous pneumothoraces. The underlying mechanism for bullae formation is uncertain, but probably relates to direct lung toxicity and repeated barotrauma as the smoker performs frequent valsalva manoeuvres in an attempt to derive a greater drug effect.

**Case presentation:**

We describe a case of probable "bong lung" occurring in a 23-year-old Caucasian man with cystic fibrosis who had a history of recurrent pneumothoraces and unusual findings on sputum cytology.

**Conclusion:**

Our case highlights the importance of questioning young adult cystic fibrosis patients about illicit drug use and the utility of sputum cytology and computed tomography scanning when patients present with pneumothoraces and deteriorations in clinical status.

## Introduction

Marijuana smoking has recently been identified as a risk factor for bullous lung disease and the occurrence of recurrent pneumothoraces [1,2]. Upper lobe lung deposition of hot particulate matter following inhalation stimulates an inflammatory response that is characterised by the accumulation of carbon-laden alveolar macrophages (AM) and polymorphonuclear cells (PMNs) in the airway lumen and lung parenchyma [3]. Marijuana suppresses the ability of AM to phagocytose pathogens with an increased risk of airway infection and lung abscesses [4,5]. There are also suggestions that marijuana smoking increases the risk of lung cancer, although this is as yet not definitely established [6,7].

We describe the case of a 23-year-old Caucasian man with cystic fibrosis (CF) with recurrent pneumothoraces, which were most probably due to "bong lung" and the presence of unusual findings on sputum cytology.

## Case Presentation

A 23-year-old Caucasian man with CF was admitted with a one-week history of pleuritic chest pain, increased cough and sputum purulence, accompanied by some minor haemoptysis. Lung function had deteriorated; FEV_1 _of 2.01L (42% predicted) compared to 2.76L (57% predicted) when well. On examination, he was clubbed and malnourished (body mass index: 18), but not cyanosed. Auscultation of his chest revealed widespread inspiratory crackles over both upper lobes. A diagnosis of an infective exacerbation of his bronchiectasis was made. He was continued on intravenous ceftazidime and tobramycin, regular physiotherapy and nutritional supplementation.

Past medical history consisted of pancreatic insufficiency and chronic airway sepsis related to *Pseudomonas aeruginosa *infection. Diagnosis had been made at birth and he possessed the ΔF508/1898 + G → CF gene mutation. He was known to have established osteoporosis and significant gastro-oesophageal reflux. Over the preceding two years, he had been admitted to a hospital on 12 occasions with acute exacerbations of his CF lung disease and during this time period, his forced expiratory volume in one second L (FEV_1_) had deteriorated from 3.17 L (74% predicted) to 2.76L (57% predicted). He also had a past history of recurrent left-sided pneumothoraces.

**Figure 1:**
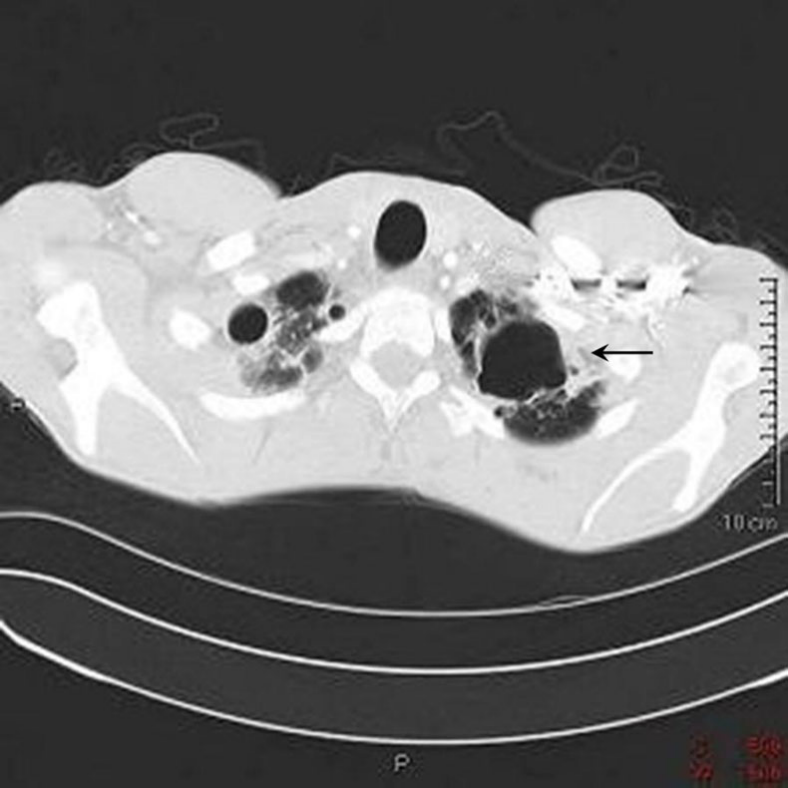
**High resolution CT scan of the patient's lungs demonstrating apical bullae**. Note the prominent bronchial arteries (arrow).

**Figure 2:**
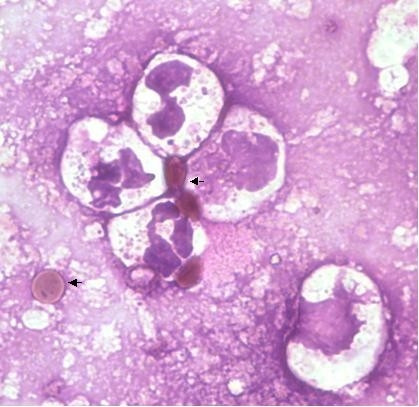
**Sputum cytology using Giemsa stain (x100 magnification and oil immersion lens)**. Note the brown oily material (arrows) and surrounding PMNs.

During the admission, he developed spiking fevers and complained of worsening pleuritic pain. A Computed Tomography (CT) pulmonary angiogram was performed to look for pulmonary emboli. The CT scan showed no emboli, but demonstrated large bilateral upper lobe lung bullae, more prominent on the right side with characteristic bronchiectatic changes elsewhere (Figure 1). Sputum microscopy revealed the expected predominance of PMNs, but also droplets of oily brown material embedded in mucus. PMNs were observed containing vacuoles full of this brown-pigmented substance and elsewhere, these droplets could be seen surrounded by palisades of PMNs (Figure 2). On closer questioning, the patient admitted to several years of marijuana smoking through a bong. He denied use of tobacco. A provisional diagnosis of "bong lung" complicating severe CF bronchiectasis was made. The patient made a slow recovery over three weeks and received counselling about his marijuana use. He was discharged with an FEV_1 _of 2.33L (48% predicted).

## Discussion

Marijuana lung has been described in habitual smokers, but not previously in the setting of CF. Our patient presented with frequent exacerbations on the background of recurrent spontaneous pneumothoraces and a rapid deterioration in lung function. He was found to have large apical bullae on high-resolution computerized tomography (HRCT) scanning, similar to those typically observed in marijuana smokers. To the best of our knowledge, this is the first reported case of bong lung in CF, but the prevalence of marijuana use in CF has been reported to be as high as 20% suggesting this may be a potentially under-diagnosed condition [8]. Bong lung is worth considering in CF adults, especially as pneumothorax is such a relatively common complication of the disease with three quarters of cases occurring in patients aged over 18 years old [9].

Our case was particularly notable for the novel appearances on sputum microscopy of palisades of PMNs surrounding and trying to engulf/phagocytose droplets of marijuana. There have been no previous reports of airway PMNs containing unusual inclusion bodies in marijuana smokers, but enhanced PMN recruitment and activation in CF related to marijuana inhalation may exacerbate the already over-exuberant innate immune response and promote further lung injury.

## Conclusion

Our case highlights the importance of considering drug abuse/marijuana use in CF patients, particularly in the context of atypical paraseptal bullae, recurrent pneumothoraces, accelerated decline in lung function and repeated admissions with increasing airway sepsis. Consideration should be given to sputum cytology and CT scanning when CF patients develop an unexplained deterioration in respiratory status.

## Abbreviations

AM: alveolar macrophages; CF: cystic fibrosis; CT: Computed Tomography: FEV_1 _:forced expiratory volume in 1 second L; HRCT: High Resolution Computed Tomography; PMNs: polymorphonuclear cells.

## Competing interests

The authors declare that they have no competing interests.

## Authors' contributions

DR was the consultant physician caring for the patient at the time of presentation and diagnosis, and ZG was a medical student attached to the respiratory unit at the time. ZG identified the uniqueness of the case and wrote the first draft of the case report. DR contributed to the writing of the case report as did his colleagues RWB, KM and RH. KM was responsible for interpretation of the sputum cytology and RH interpreted the radiology. JH elucidated the history of substance abuse and contributed to the review of the manuscript. All authors have read and approved the final manuscript.

## Consent

Written informed consent was obtained from the patient for publication of this case report and any accompanying images. A copy of the written consent is available for review by the Editor-in-Chief of this journal.
